# The Amazing Acrobat: Yeast’s Histone H3K56 Juggles Several Important Roles While Maintaining Perfect Balance

**DOI:** 10.3390/genes12030342

**Published:** 2021-02-25

**Authors:** Lihi Gershon, Martin Kupiec

**Affiliations:** The Shmunis School of Biomedicine & Cancer Research, Tel Aviv University, Ramat Aviv 69978, Israel; lihigers@mail.tau.ac.il

**Keywords:** chromatin, acetylation/deacetylation, genome stability, DNA replication, DNA repair, Hst3, Hst4

## Abstract

Acetylation on lysine 56 of histone H3 of the yeast *Saccharomyces cerevisiae* has been implicated in many cellular processes that affect genome stability. Despite being the object of much research, the complete scope of the roles played by K56 acetylation is not fully understood even today. The acetylation is put in place at the S-phase of the cell cycle, in order to flag newly synthesized histones that are incorporated during DNA replication. The signal is removed by two redundant deacetylases, Hst3 and Hst4, at the entry to G2/M phase. Its crucial location, at the entry and exit points of the DNA into and out of the nucleosome, makes this a central modification, and dictates that if acetylation and deacetylation are not well concerted and executed in a timely fashion, severe genomic instability arises. In this review, we explore the wealth of information available on the many roles played by H3K56 acetylation and the deacetylases Hst3 and Hst4 in DNA replication and repair.

## 1. Introduction

Each time a cell divides, it must, within a short time span, make a new copy of its whole genome. In eukaryotic cells, the DNA is wrapped around nucleosomes, the basic unit of chromatin. The nucleosomes contain a pair of each type of histones: H2A, H2B, H3, and H4, and spool 147 base pairs of DNA around them [1]. These histones carry various modifications (acetylation, ubiquitylation, SUMOylation, phosphorylation, etc.) at various sites, and together they create the epigenetic memory, which must be duplicated to daughter strands as faithfully as the genome itself [2]. In this review, we concentrate on the roles played by acetylation of histone H3 at lysine 56, in particular in the yeast *Saccharomyces cerevisiae*, for whom a wealth of information has been gathered in the last decade.

As cells replicate their DNA into two daughter strands, a complicated process of removing old nucleosomes from the chromatin located ahead of forks and transferring them to behind the forks takes place. However, the old nucleosomes are numerically insufficient for the task of establishing chromatin on the two daughter strands (as there is now twice the amount of DNA), and so new histones are made and incorporated together with old histones onto the nascent daughters. These new H3 histones are acetylated at lysine 56 following a chain of handoffs starting with the histone chaperone Asf1 and ending with chromatin remodelers [3,4,5,6,7].

Acetylation of histone H3 at lysine 56 (hereafter referred to as H3K56Ac) is a modification that plays a significant role in cell survival and DNA transmission fidelity, but is poorly understood even today. Regardless of the stage of the cell cycle in which histone exchange is carried out, new H3 histones will always be acetylated on lysine 56. In cells arrested at the G_1_ stage of the cell cycle, for example, a galactose-induced histone H3 protein showed Asf1-dependent acetylation (see below), indicating that H3K56Ac is not limited to S-phase [8]. 

The histone tails are the first 20–30 amino acids protruding from the nucleosome surface [9], and while most histone modifications occur at the exposed N-terminal tails, lysine 56 is unique in that it engulfs the last 10 bases of the DNA that enters and exits the nucleosome. The acetyl group also makes water-mediated bonds with the DNA phosphate backbone [10]. This particular position puts H3K56Ac in an ideal location to facilitate DNA accessibility to various proteins. Consistent with this notion, acetylation of K56 increases spontaneous DNA breathing, doubles the fraction of nucleosomes with unwrapped DNA at the end position, and quadruples the unwrapping internally to K56 [11]. In vitro experiments showed that upon mimicking of H3K56 acetylation using the K56Q allele, there was a two-fold increase in mobility [12]. It was further found that K56Ac nucleosomes allow for more access to micrococcal nuclease [13] and that DNA binding to H3K56Ac-H4 tetramer was 15-fold weaker than to unmodified H3-H4 [3]. All these lines of evidence support a model in which acetylation of H3K56 enables accessibility to the DNA. 

While this review is concentrated on histone H3 lysine 56 acetylation, it is not the only modification that histones H3-H4 undergo that is important for deposition. K5, K8, K12, and K91 acetylation on histone H4 by Hat1 [14] are also markers of newly synthesized histones and are implicated in replication-coupled nucleosome assembly [15]. Furthermore, other than K56, H3K115, and K122, as well as H4 K31, S47, and K79 are also positioned for interactions with the DNA, although not at the entry and exit sites [16]. 

## 2. From Acetylation to Incorporation

### 2.1. Asf1

**Figure 1:**
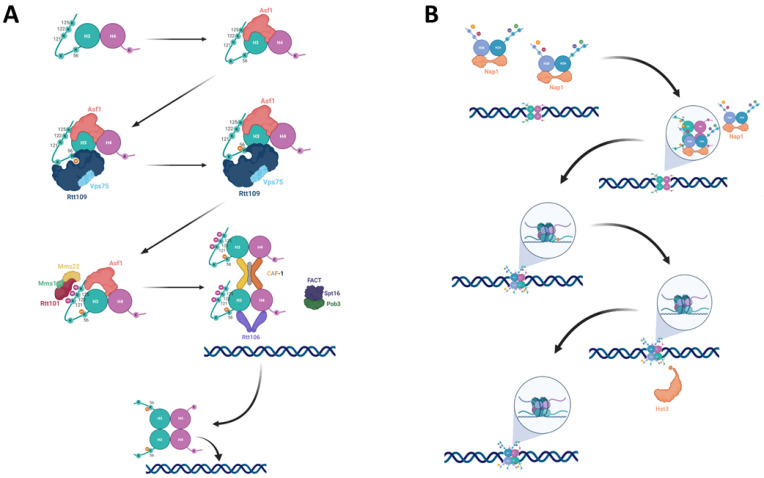
Nucleosome assembly with H3 K56-acetylated histones: (**A**) The newly synthesized H3-H4 dimer is bound by Asf1, which presents it to Rtt109. Rtt109, together with Vps75, acetylates H3 at lysine 56. The H3K56Ac-H4 dimer’s affinity to Rtt101 increases, leading Rtt101-Mms1-Mms22 to ubiquitylate H3 at K121, K122, and K125 of H3, and driving the dissociation of H3K56Ac-H4 from Asf1 and its handoff to the chromatin remodelers CAF-1, Rtt106 and FACT. (**B**) Following the incorporation of a tetramer containing two H3K56Ac-H4 dimers, two Nap1 proteins bring H2A-H2B dimers to form an octamer. Following the formation of the octamer Hst3 then deacetylates the nucleosome at position K56 of H3.

Asf1 is a histone chaperone that binds H3-H4 dimers, and in fact in mammals all histones not bound by DNA appear to be bound to Asf1 (Figure 1A) [3,14]. In addition to the deposition of histones during DNA replication, histones are dynamically exchanged as a consequence of processes such as transcription and DNA repair (replication independent nucleosome assembly). Asf1 is the only chaperone that functions during both of these processes. During replication it collaborates with the CAF-1 complex [4] while replication-independent histone deposition also requires the HIR proteins (composed of Hir1, Hir2, Hir3, and Hpc2) [5]. In addition, Asf1 is capable of mediating chromatin disassembly as well as chromatin reassembly during transcription [17,18]. The binding of Asf1 to a H3-H4 dimer prevents it from tetramerizing prematurely. This may explain how Asf1 can also play a role in chromatin disassembly [19]. 

### 2.2. Rtt109

Following binding to H3-H4, Asf1 can now deliver the histones to the acetyltransferase Rtt109 (mammalians CBP) for acetylation at lysine 56 of histone H3 (Figure 1A) [6,7]. In addition, lysines 14 and 23 of the same histone are also acetylated by Rtt109 [20], while lysines 9 and 27 may be targeted by both Rtt109 and Gcn5 for acetylation [3]. Rtt109 works together with the NAP histone chaperone Vps75, which aids in Rtt109’s nuclear import [21] and elevates the K_cat_ of Rtt109 [22,23]. In order to perform its role, Rtt109 first undergoes auto-acetylation at lysine 290. This auto-acetylation causes slight structural changes in Rtt109 that improve its affinity towards acetyl CoA; this, in turn, stimulates its histone acetyltransferase activity [24]. While Rtt109 can acetylate several residues on H3, it appears that Asf1 chaperoning of the H3-H4 dimer and its unwinding of the α helix present at the N-terminus region (αN) of histone H3 alters the Rtt109-Vps75 complex’s selectivity towards K56. Rtt109’s lysine binding channel brings the ε-amino group of H3 only 4Å away from the sulfhydryl group of acetyl-CoA, enabling the transfer of the acetyl to K56. Surprisingly, this interaction also requires direct binding between Rtt109 and the central helix of H3, which is some distance from lysine 56 of H3 [25].

### 2.3. Rtt101

Once H3 is acetylated, the H3K56Ac-H4 dimer should now be handed off to the chromatin remodelers in order to be incorporated into the assembling chromatin [13,26]. However, Asf1, CAF-1, and Rtt106 bind H3-H4 with similar affinity, and, therefore, a simple transition cannot occur [27]. Rtt101 is a Cul4 E3 ubiquitin ligase that works together with the E2 subunit Cdc34 and the RING finger protein Hrt1, as well as the adapter subunits Mms1 and Mms22 [28,29]. It was found that H3K56 acetylation promotes ubiquitylation by Rtt101 of lysines 121, 122, and 125 of histone H3. The ubiquitylation sites are located at the C-terminal helix of H3, close to the binding interface between Asf1 and H3 (Figure 1A). This position is ideal for bringing about the dissociation of the Asf1-H3K56Ac-H4 complex. Since *Δrtt101* strains exhibit normal levels of H3K56Ac, it has been concluded that Rtt101 must act downstream of the acetylation process itself [30].

### 2.4. Chromatin Remodelers

With the dissociation of the Asf1-H3K56Ac-H4 complex, the histone dimer is now handed off to the various chromatin remodelers, namely Rtt106, FACT, and CAF-1 (Figure 1A). CAF-1 recognizes H3K56Ac-H4 dimers and binds to them with higher affinity than to unacetylated histones. It is also responsible for the tetramerization of two H3K56Ac-H4 dimers prior to their deposition on the DNA [31]. Rtt106 also binds with high affinity to both dimers and tetramers containing H3K56Ac [32]. In addition, CAF-1’s binding to H3K56Ac enhances CAF-1’s interaction with PCNA, leading to histone deposition at the vicinity of the replication fork [33]. In agreement with Cac1 and Rtt106 having high affinity for acetylated H3K56, mutations in Asf1 and Rtt109 that abolish the acetylation on K56 also reduce the amount of H3 that co-purifies with either Cac1 or Rtt106 [33]. 

Recently, FACT has also been implicated in this process. FACT is a two-subunit complex, containing the non-essential Pob3 and essential Spt16 proteins. FACT is ubiquitylated by Rtt101 in order to localize to the replisome [28]. FACT co-purifies with both H3K56Ac and unmodified H3 histones, as well as the Cac2 subunit of CAF-1 and with Rtt106. It was found that Rtt106’s interaction with FACT is decreased when H3 is not K56 acetylated, suggesting that FACT, CAF-1, and Rtt106 may work in a coordinated fashion. Cells lacking both Cac1 and Rtt106 are still viable, hinting that other factors participate in histone deposition onto the DNA. FACT’s position at the replication fork, and the finding that an H3 binding-deficient allele of FACT in *Δrtt106* or *Δcac1* strains reduces nucleosome assembly, further suggest that FACT takes part in the chromatin deposition of K56 acetylated histones [33,34]. 

### 2.5. Nucleosome Assembly

Following the tetramerization of H3-H4 dimers, the tetrasome is deposited onto the DNA. H2A-H2B are deposited onto the DNA as dimers by Nap1 [35]. The dimers are then tethered, each to a different half of the H3-H4 tetramer, forming an octamer (Figure 1B) (reviewed in [36]). The H2A-H2B dimers and the H3-H4 tetramers do not have a preference for associating with their like (i.e., parental histones with parental histones) and instead form hybrid octamers to ensure that epigenetic information is inherited by the two forming double-stranded DNA molecules that will constitute the sister chromatids [37]. 

In addition to the new histone deposition, parental H3-H4 histones are transferred from the front of the fork to behind it by a still mysterious mechanism involving the Mcm2 component of the MCM helicase, which creates an H3-H4 mediated contact with Asf1 for this purpose [38]. Dpb3 and Dpb4, two factors connected to the replication fork and DNA polymerase ε, appear to aid in this process. Their activity creates a slight bias for parental histones towards assembling onto the lagging strand versus the leading strand [39]. Following the assembly of the nucleosome, chromatin remodeling factors, such as the ISWI-containing ACF complex, rearrange the nucleosomes on the DNA at even spacing (reviewed in [36]). As soon as enough DNA has been generated by the replisome, it is spooled onto the nucleosomes in a 1¾ left-handed superhelical turn that stretches 147 base pairs of DNA [1]. 

## 3. Histone Deacetylation

The acetyl moiety of H3K56 is removed by Hst3 and Hst4, two redundant NAD^+^-dependent histone deacetylases from the sirtuin family (Figure 1B). Other members of the family include Sir2, the founding member that gave the HST family its name (Homolog of Sir Two) as well as Hst1 and Hst2. However, despite the homology, these proteins are implicated in different processes and do not share overlapping functions. Only Hst3 and Hst4 are able to completely and globally deacetylate histone H3K56 [40]. H3K56 acetylation is cell cycle dependent, and in unperturbed cell cycles, it accumulates during the S phase and is removed at G_2_/M by Hst3 and at M/G_1_ by Hst4. If the cell cycle is perturbed (for example, by DNA damage), K56 may remain acetylated for longer time as the cells are arrested for the duration of the perturbation (see more details below) [13].

### 3.1. Hst3

Hst3 plays a more prominent role in the deacetylation of K56 than Hst4, as in cells lacking Hst3 there is accumulation of H3K56Ac on the histones throughout the cell cycle. In contrast, strains deleted for *HST4* retain WT levels of acetylation as the cell cycle progresses [41]. In agreement with the role of H3K56Ac in marking newly synthesized H3 histones, after one cell cycle in the absence of Hst3, ~50% of the histones carry this mark [42].

No evidence was found for a link between Hst3 and any of the acetylation machinery proteins and further, de novo expression of Hst3 in arrested cells outside of S phase produced rapid loss of any acetylated K56, implying that Hst3 acts by promoting the deacetylation of H3K56, rather than suppressing the acetylation machinery [41].

Under normal conditions, Hst3 is characterized by a sharp rise at the G_2_/M stage of the cell cycle, similar to other proteins that behave like the Clb2 cyclin [43]. The C-terminus of Hst3 (clustered around threonines 380/384 and serines 420/421) contains minimal consensus sequences for phosphorylation by the cyclin-dependent kinase (CDK) Cdc28, and both of them are needed for complete degradation. Mutating these residues led to a higher abundance of Hst3, suggesting that Cdc28, together with components of the SCF ubiquitin-ligase complex, the E2 subunit Cdc34 and the F-box protein Cdc4, are needed for the degradation of Hst3 before the start of mitosis [44]. Although cell cycle regulated, Hst3’s degradation by Cdc28 does not require the Anaphase Promoting Complex (APC). As it seems surprising that Hst3 levels rise and fall together with those of Clb2, which is in charge of sending it to degradation, it was proposed that differences in the kinetics of accumulation serve to prevent premature Hst3 degradation by Cdc28. The sharp rise in G_2_/M in the levels of Hst3 means that it takes some time for the Cdc28 protein to accumulate sufficiently to target all Hst3 molecules, and this can ensure that by the time of its degradation, Hst3 has managed to deacetylate H3K56 throughout the whole genome [44]. Furthermore, while T380/384 and S420/421 contain minimal consensus sequences for Cdc28, their sequence also matches exactly that of the kinase Mck1. It is indisputable that these sites contribute to Hst3’s degradation, since mutating them increases the half-life of Hst3 from 8 minutes to 45 minutes. Thus, it appears that Hst3 levels are regulated by both Cdc28 and Mck1. Importantly, cell cycle arrest at G_2_/M by a galactose inducible Swe1 protein, which inhibits Cdc28, still resulted in retention of H3K56Ac, showing that Mck1 alone (without CDK activity) is able to handle Hst3 degradation [45,46]. It is, therefore, possible that the two kinases work under different conditions, which are yet to be defined [47]. 

Upon activation of the S-phase checkpoint, Hst3 is under the regulation of Mec1, both transcriptionally and at the protein level. Both Mec1 and Rad53 were shown to prevent accumulation of Hst3 transcripts [42]. In addition, the Hst3 protein is poly-ubiquitylated and degraded by the proteasome. Mec1 mediates a decrease in the half-life of Hst3 from 8–9 minutes in unperturbed cell cycle to 3.5 minutes under stress conditions [42,48]. 

### 3.2. Hst4

Hst4 works at a different phase of the cell cycle from Hst3, and acts as a backup in case Hst3 has not completed its job. It peaks after Clb2 and Hst3 levels have dropped and remains expressed at high levels throughout the M to G_1_ phase, until the cells enter S-phase [42]. Despite sharing only 42% similarity with Hst3, when expressed under the promoter of Hst3, Hst4 is capable of maintaining WT levels of acetylation in the absence of Hst3 [49]. Thus, although functionally identical, the different cell cycle regulation gives Hst3 the leading role.

Hst4 has been found in a screen for Cdc4-binding proteins, suggesting that it is degraded after being ubiquitylated [44]. It is excluded from the nucleus in WT cells during the G2/M phases, consistent with its role later in the cell cycle. However, regardless of the cell cycle stage, Hst4 can be found in the cytoplasm and in mitochondria [50]. 

### 3.3. Δhst3 Δhst4

In the double mutant *Δhst3 Δhst4,* H3K56 is acetylated in all histones and throughout the entire cell cycle. This leads to a plethora of genomic instability phenotypes, which are summarized in Table 1. Many of these phenotypes are phenocopied by either nicotinamide (NAM) [51] or by introduction of an H3K56Q allele [52], both of which mimic hyper-acetylation to varying degrees. NAM is an inhibitor of deacetylases of the sirtuin family, which include Hst3 and Hst4. The H3K56R mutation, which prevents histone acetylation, can rescue most of the genomic instability phenotypes of *Δhst3 Δhst4* cells. However, a cycle of acetylation/deacetylation seems to be important: cells unable to acetylate histone H3 on lysine 56, due to deletion of Asf1 or Rtt109, or expressing the non-acetylatable allele H3K56R as the only source of histone H3, suffer genome instability phenotypes as well: susceptibility to genotoxic agents such as methylmethane sulfonate (MMS), campthotecin (CPT), and hydroxyurea (HU), delayed checkpoint activation and delayed exit from cell cycle arrest. It has been proposed that H3K56 acetylation and deacetylation is important for the chromatin unwrapping and re-constitution associated with DNA repair [53].

## 4. Hyper-Acetylation and Replication

When WT cells start DNA replication, all histones have been deacetylated by Hst3 and Hst4 in the previous cell cycle. As the fork progresses and new DNA is created, behind the fork new chromatin is formed by mixing old, unmarked histones imported from the replisome front, with newly synthesized, H3K56 acetylated histones. One of the advantages of such a layout is that it provides the ability to recognize which way is the front of the fork and which way is the back. This is important, as behind the fork the sister chromatid is being created. Being identical in sequence, sister chromatids are the best template with which to repair various DNA lesions that require a donor sequence [57].

Several lines of evidence point to the presence of H3K56Ac on the chromatin as being disruptive to the replication process. Cells that are *Δhst3 Δhst4* show synthetic lethality with *pol2-11*, a temperature-sensitive allele of Polε’s major subunit, Pol2, which lacks replicative functions at restrictive temperatures. Furthermore, merely tagging either Cdc45 (with myc) or PCNA (with HA) also leads to synthetic lethality, showing that the replication is extremely sensitive to subtle perturbations when the genome is hyper acetylated [52].

Recently it was shown that hyper-acetylation of H3K56 affects the dynamics of the overall replication, by affecting firing of origins. In the presence of hyper-acetylation, there is constant activation of Rad53, which must be counteracted by the Dun1 checkpoint protein kinase for cells to survive [58]. The activation of Rad53 by the hyper-acetylated state of the chromatin (which is discussed extensively below in the context of checkpoint activation) causes Rad53 to inhibit late firing origins, and this in turn forces forks to traverse longer distances in order to complete genome duplication. Such a state has previously been proven to lead to fork stall and collapse [59], and indeed, in cells with hyper-acetylated genome such a situation leads to lethality.

PCNA loading appears to play an important role in cells with hyper-acetylation of H3K56. As mentioned, *Δhst3 Δhst4* cells are thermosensitive (TS) and sensitive to genotoxic agents. However, various factors are able to abolish these phenotypes without changing the acetylation state of H3K56. Over-expression of the essential protein Rfc1, which encodes the large subunit of the Replication Factor C complex, suppresses the TS phenotype and, to a lesser degree, the genotoxic sensitivity of *Δhst3 Δhst4*. Similar phenotypes are observed when deleting each of the Replication Factor C-Like Complexes (RLCs) proteins Elg1, Rad24, and Ctf18 [52]. The fact that eliminating other users of Rfc2-5 subunits or providing more of the canonical Rfc1 subunit is enough to overcome the TS phenotype of *Δhst3 Δhst4* points to a viability-promoting role performed by the Rfc1-loaded PCNA [52]. Indeed, it is known that Elg1, for example, unloads PCNA [60,61] and, therefore, its presence might mitigate PCNA’s role in promoting survival of *Δhst3 Δhst4*. 

Several components of the replisome have been shown to interact with H3K56 acetylated histones. Rrm3 is a helicase that travels with the fork and is required for the replisome to pass through protein-DNA complexes [62]. Deletion of Rrm3 was found to suppress the TS phenotype of *Δhst3 Δhst4* [58]. However, mutations in the acetylation machinery (Asf1, Rtt109, Rtt101, Mms1, and Mms22) together with deletion of Rrm3 are all lethal [63]. The fact that perturbations in H3K56 acetylation and deacetylation cycle are both detrimental may mean that Rrm3’s activity may be context (i.e., histone modification) dependent. 

Ctf4 is a replication fork-associated protein that functions in chromosome segregation as well as in DNA replication by coordinating between DNA synthesis and unwinding, and specifically by coupling Polα to the MCM helicase at the lagging strand through its interaction with the GINS complex [64]. Deletion of *CTF4* is able to suppress the TS and HU-sensitivity phenotype of *Δhst3 Δhst4* mutant cells [52]. Furthermore, while mutating the acetylation machinery in the absence of Rrm3 resulted in lethality, additional mutation in Ctf4 (e.g.,: *Δasf1 Δrrm3 Δctf4*) rescued this phenotype [63]. While it may appear that Ctf4’s role is opposing that of Rrm3, it is possible that its detrimental activity is connected to its ability to incorporate Polα into the replisome [65]. As DNA Polα is essential but Ctf4 is not, replication can take place even when Polα is not efficiently coupled to the helicases. It is possible that in such a state replication dynamics are more accommodating to hyper-acetylated chromatin, which will explain Ctf4’s ability to suppress both the lethality of Rrm3 and the TS phenotype of *Δhst3 Δhst4*.

It has been speculated that dormant origins are of particular importance in cells with hyper-acetylation of H3K56. Dormant origins are origins that have been primed to fire, but statistically fire only sporadically rather than on each cell cycle and in each yeast cell. They are thought to be a failsafe mechanism in case of fork collapse [66]. Cells deleted for Mrc1, which is found both at the checkpoint and at the replisome [67], showed replication from a greater number of dormant origins than the WT [68]. This over-abundance of firing leaves Mrc1 mutants with very few dormant origin reserves in case of fork stall and collapse. Cells deleted for Mrc1 are also synthetic lethal with *Δhst3 Δhst4*’s replicative function [58]. These cells experience slow replication kinetics despite the over-abundance of firing, but are able to survive if factors, which limit replication elongation, such as Ctf18 and Rtt101, are deleted [69]. Rrm3, which also removes the MCM2-7 subunits from dormant origins when the CMG helicase reaches them [66], has a detrimental effect on *Δhst3 Δhst4* and its deletion leads to the suppression of the TS phenotype of *Δhst3 Δhst4*, as was stated above. Since MCMs are not loaded onto the chromatin once the cells exit G_1_ [70], it is possible that Rrm3’s activity is detrimental because it causes an irreversible reduction in the number of dormant origins available to aid replication in the event of fork stalling.

**Figure 2:**
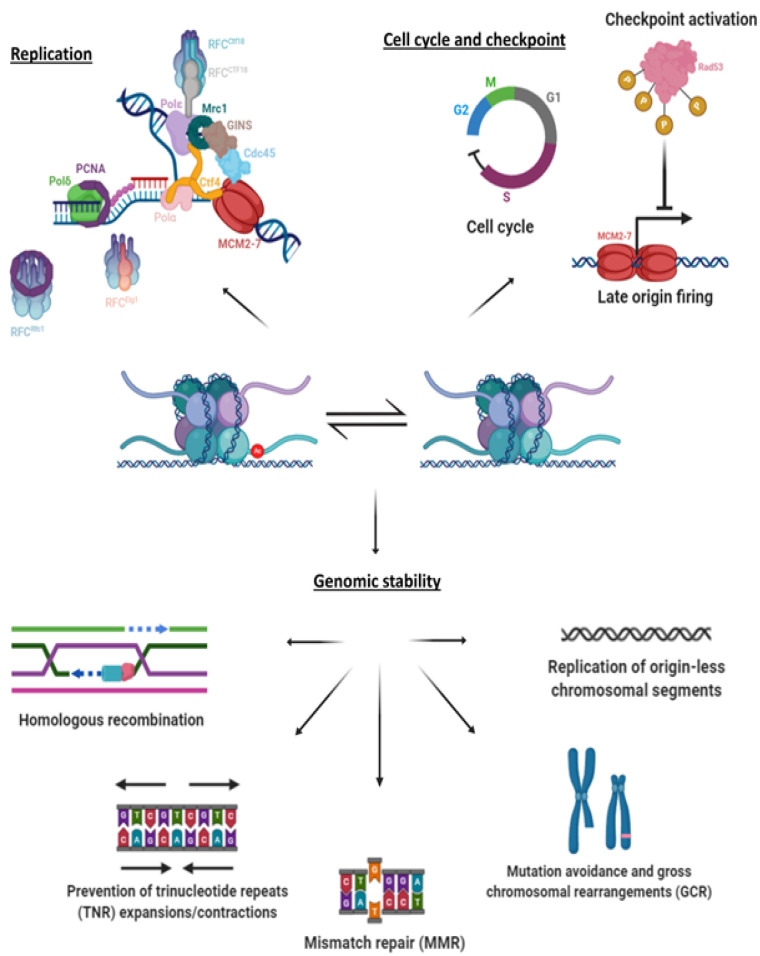
A summary of the different roles of H3K56Ac: H3K56Ac has been implicated in a variety of processes, related both to replication and to genome stability. Of these processes, evidence exists that ties H3K56Ac to homologous recombination repair pathways, in particular strand invasion and break induced repair (BIR). In addition, there is an effect on mismatch repair, expansions and contractions in the number of trinucleotide repeats, maintenance of origin-less chromosomal segments, and the prevention of chromosomal abnormalities such as mutations and rearrangements. H3K56Ac is also connected to replication, and to firing of origins and cell cycle progression. See text for more details.

The sum of these results (Figure 2) brings into focus the importance of maintaining strict and timely control over acetylation and deacetylation of H3K56. Although it is only one out of many posttranslational modifications, acetylation of K56 occurs at a crucial location on the nucleosome and has many implications on replication. 

## 5. Hyper-acetylation and Genome Stability

The fact that cells suffering either hypo- or hyper-acetylation of H3K56Ac exhibit genome instability, coupled with the rapid degradation of Hst3 upon checkpoint activation, suggests that the presence of acetylation and its removal in an orderly manner contributes to DNA repair or plays a role in the avoidance of genomic mutations/rearrangements. Any repair of the DNA following the passage of the fork is inevitably done in the context of the chromatin and, thus, necessitates the removal of nucleosomes at the affected area and their subsequent reinstating, in a model known as “Access-Repair-Restore” (ARR) [71]. The old histones are sent to proteasome-mediated degradation, and, therefore, cannot be of use in the reinstating process [72]. This is a very likely reason for why K56 acetylated H3 histones were observed to be prevalent at sites of DNA damage [48]. However, this alone cannot cover the scope of the effects on repair and genome stability phenotypes observed in mutations of components of the acetylation pathway, as will be discussed here (Figure 2). 

It is important to stress that the importance of H3K56 acetylation for genome stability is limited to S phase, excluding its involvement in repair pathways that are mainly active at the G_2_/M phase. For example, Rtt109 mutants do not experience sensitivity to ionizing radiation (IR) or to its mimicking agents such as phleomycin and bleomycin [6]. In addition, Hst3 is not downregulated when Double Strand Breaks (DSBs) are induced by said agents [42]. This is because repair of IR-induced DNA damage occurs at the G_2_ phase of the cell [73].

### 5.1. Checkpoint Activation and Recovery

When cells are challenged with exogenous or endogenous stresses, be it replication-transcription collisions or exposure to genotoxic agents creating DSBs, the immediate consequence is the transient slowing or stalling of replication fork progression or of DNA synthesis entirely. In order to protect stalled forks and ensure that replication can recommence, as well as to avoid copying damaged templates and carrying mutations on to the next generation, cells activate a response called the S-phase checkpoint [74]. This response is composed of several components, which sense the damage and transduce signals to evoke a suitable response. The signal that activates the checkpoint response is thought to be single stranded DNA (ssDNA) coated with RPA, a complex that is usually only transiently made, but accumulates if the leading and lagging polymerases become disconnected, or following resection of double stranded DNA (dsDNA) [4,75,76]. Following the formation of the ssDNA, a series of phosphorylation events takes place that is aimed at amplification of the checkpoint signal, in order to induce cell cycle arrest and repair. Mec1 phosphorylates histone H2A on serine 129 (γ-H2A, mammalian H2AX) in a large domain around the site of the DNA damage or problem [67,77]. The signal is then transduced through the adapter proteins Mrc1 (via the DNA Replication Checkpoint, or DRC) and Rad9 (via the DNA Damage Response, or DDR), which promote the phosphorylation and activation of the kinase Rad53 (mammalian CHK2). Rad53 phosphorylates a large number of proteins, mediating the upregulation of dNTPs, inhibition of late firing origins, fork stabilization, and the induction of repair genes [74,78]. 

The S-phase checkpoint can be inactivated either when the damage has been repaired (recovery) or in the presence of persistent DNA damage (adaptation). In the first case, the original lesion is repaired, and inhibitors of the checkpoint proteins can suppress its activation [79]. For example, the PP4 complex, composed of Pph3, Psy2, and Psy4, is needed for the de-phosphorylation of Rad53, which is thought of as the indication that the checkpoint has been deactivated [80]. 

Deletion of the checkpoint gene *RAD53* rescues the TS phenotype of *Δhst3 Δhst4*, showing that the activity of the checkpoint has detrimental consequences for cells experiencing hyper-acetylation of K56, possibly due to its cell cycle arrest function. However, it appears that the DDR branch of the checkpoint, activated by Rad9, is particularly involved in the adverse response to hyper acetylation [52]. Deletion of *PPH3* caused lethality in *Δhst3 Δhst4* cells, while deletion of *RAD9* improved cell viability [81]. This shows that preventing Rad9-mediated Rad53 activation improves viability under hyper-acetylation conditions. In agreement with this, many of the spontaneous TS suppressors of *Δhst3 Δhst4* mutants affect either γ-H2A accumulation or Rad53 phosphorylation [81]. It is of note that Mrc1, the other adapter of Rad53, is vital for the viability of *Δhst3 Δhst4* cells, as its deletion leads to cell death. However, Mrc1 is a multifunctional protein and, using separation of function alleles, it was discovered that Mrc1 is needed due to its replicative function, rather than its ability to induce the checkpoint.

Cells lacking either Rtt109 or Asf1 are capable of executing all steps needed for repairing a single HO-induced DSB, but are incapable of reassembling chromatin following these processes. In these cells, the DNA checkpoint remains active following repair and re-synthesis, and the cells are delayed in re-entering the cell cycle in comparison to WT. Chromatin reassembly, however, was fully restored in a strain harboring both *ASF1* deletion and H3K56Q, showing that rather than Asf1, it is the H3K56 acetylation per se that is needed [82]. Another study found that Rtt101 is also needed for recovery from checkpoint activation [83]. This means that H3K56Ac may act as a mark for the completion of repair and chromatin reassembly following DSBs, and this signal seems to be important for checkpoint recovery. Supporting this hypothesis, Hst3 is under the regulation of the S-phase checkpoint, and upon checkpoint activation undergoes proteasome-mediated degradation, causing persistent acetylation for the duration of the checkpoint activation. Indeed, in *Δhst3 Δhst4* cells, there is constitutive Rad53 phosphorylation, which is thought to reflect the genomic instability of this mutant, as it is accompanied by persistent γ-H2A signal [52]. 

### 5.2. Mutations and Genomic Instability

Base mis-pairs, as well as small insertions or deletions occurring during the process of DNA replication, which escape the proofreading abilities of the DNA polymerases, are handled by the Mismatch Repair pathway (MMR). This pathway can recognize the mismatch using the Msh2-Msh6 (homologues to MutSα), or the Msh2-Msh3 (homologues to MutSβ) protein complexes that bind to the mismatch and to the replicative clamp PCNA. Following the binding, these proteins bind to the Mlh1-Pms1 protein complex (homologues to MutLα), which initiates an ATP-dependent nicking of the daughter strand around the damage area. These proteins then recruit Exo1 to excise the area with the mismatch and fill the gap using DNA polymerase δ [84]. This process is spatially and temporally linked to the replication itself [85], which raises the possibility that the MMR is connected to nucleosome deposition. However, while MutSα is able to pull down an Asf1-H3-H4 complex by interacting with the histone components in this complex [86], its binding to mismatches incorporated on nucleosomes in vitro was decreased [87]. 

*Δhst3 Δhst4* strains suffer from elevated rates of mutation and gross chromosomal rearrangements (GCR). Analysis of the sequence at the break area of the GCRs revealed that most of these events were chromosomal translocations with a micro-homology of 3–12 bases at the break junction [88]. Interestingly, ectopic gene conversion, single strand annealing, and mating type switch recombination were not impaired in this strain. Analysis of the types of mutations observed in *Δhst3 Δhst4* strains revealed an elevation in 1-bp insertions/deletions, medium-sized deletions (favoring direct repeats), complex mutations, as well as transitions and transversions at a rate and distribution similar to those of strains carrying a mutation in *MSH2* [55]. This suggests that the mutations created in *Δhst3 Δhst4* are created by a mechanism involving the mismatch repair system [55]. Indeed, further results confirmed that Hst3 and Hst4 are important for mutation avoidance mechanisms that cooperate with MMR components, as well as with the proofreading activities of the replicative DNA polymerases [55].

Although hyper-acetylation increases mutation rates, lack of acetylation has a similar (if somewhat reduced) effect: both H3K56R and *Δrtt109* strains show elevated mutation rates that are synergistic with mutations in the mismatch repair system. These results point to a role for H3K56 acetylation in promoting replication fidelity, which is dependent on precise control of acetylation and deacetylation. While it is apparent that these mutations occur in S phase, it is possible that the elevated level of mutations is a result of pre-mutagenic lesions as a consequence of having K56 acetylation also at the G_1_ phase in *Δhst3 Δhst4* strains or of lack of acetylation in the previous S-phase due to deletion of *RTT109* or *ASF1* [55].

Heteroduplex rejection is a process by which recombination intermediates that are recognized by the MMR are unwound by the Sgs1-Top3-Rmi1 helicase–topoisomerase complex [89]. This is a double edged sword: if rejection does not occur, the MMR would initiate repair of the mismatch, which can be mutagenic, but if rejection is too stringent, the result will be DSB that remain unrepaired (reviewed in [90]). It was recently shown that *Δhst3 Δhst4* mutants show an eight-fold decrease in heteroduplex rejection, and lower rates of rejection were also observed for Asf1 and Rtt109 mutants, as opposite to higher than WT rates observed for Cac1 and Rtt106 mutants [91]. This result might provide an alternative explanation for the formation of the mutations in *Δhst3 Δhst4* mutants, and the need for precise acetylation and deacetylation.

H3K56 was also shown to be important for maintaining large chromosomal regions that lack firing origins. Cells avoid having large gaps between origins, as this may increase the odds of fork stalling, which could lead to under-replication of the DNA in the region. This feature of regularly distributed origins is evolutionary conserved between distant yeast species [92], such as between *S. cerevisiae* and *K. lactis*, two species that diverged prior to the whole genome duplication [93]. In addition, the largest origin-less region observed in the genome is significantly smaller than what would be created stochastically [92]. These data indicate that the deleterious consequences of uneven origin distribution have shaped the way the genome is replicated over the course of evolution.

It has been experimentally proven that although several chromosomes contain large origin-less segments, they can segregate properly in most of the cell divisions. However, these chromosomes are sensitive to subtle replication perturbations and to chromatin structure due to the need for forks to traverse longer distances [59]. Deletion of *HST3* elevated the levels of instability of a chromosome with such an origin-less segment during S-phase. This was examined by deleting the five most efficient origins on chromosome III, producing a large inter-origin region into which an *ADE2* gene was cloned and testing for red-white colony sectoring. In *Δhst3*, the ability of cells to maintain this fragment was extremely reduced (the chromosome was lost in 10^−5^ of the cells in the WT, compared to 10^−2^ in *Δhst3*) and a double mutant *Δhst3 Δhst4* was inviable [94]. It appears that while the deacetylation of H3K56 is vital for the maintenance of such a fragment, the acetylation is less important because strains lacking Asf1 or Rtt109 showed no problems in segregating this fragment [49].

Finally, H3K56 has also been connected to the stability of repetitive sequences. These large families seem to have biological significance, as not only are they not lost during evolution, they are actively maintained. A good example of this are the repetitive sequences found in telomeres and centromeres [95]. Instability of these repeats has severe clinical manifestations, such as Huntington’s disease and Fragile X Syndrome [96]. In particular, in vitro it was observed that more natural replication pausing and fork reversal events occurred at CAG/CTG repeats, indicating that they are challenging templates for replication [97,98]. CAG/CTG repeats also interfere with Base Excision Repair (BER), Nucleotide Excision Repair (NER), and MMR repair processes [99]. Contractions and expansions of repeated DNA are the addition of more repeats, or the reduction in their number during replication, respectively. Imbalance in the mechanisms of H3K56 acetylation and deacetylation causes an increase in the loss of genetic material due to CAG/CTG contractions. Rad51-dependent contractions were observed in Asf1 and Rtt109 mutants, but not in Rtt101 mutants, linking such contractions to the acetylation of H3K56 [100]. A similar elevation in the rate of contractions was also observed in deacetylation-deficient cells [101]. Studies using ChIP analysis have shown that cells unable to acetylate H3K56 accumulate replication intermediates and lose their replisome integrity following HU treatment due to H3K56Ac dependent, CAF-1 and Rtt106 mediated, nucleosome assembly defects. Such accumulation could occur if the replication and nucleosome assembly are uncoupled, and might favor CAG/CTG contractions, showing that a tight control of acetylation is important to prevent CAG repeat contractions [101]. Nucleosome assembly occurs once enough DNA has been synthesized by the replisome. There is evidence that CAG/TCG repeats contract more frequency at the lagging strand [102]. Okazaki fragments need to undergo maturation before such a process occurs, making them susceptible to said contractions [103]. It is possible that in the absence of the acetylation, there is a direct effect on nucleosome assembly that is worse for the lagging strand, which is more prone to secondary structures formation and, therefore, to CAG/CTG contractions, while in the case of hyper acetylation, the contractions will occur at the S-phase following the hyper acetylation event [101,104,105].

### 5.3. Repair Pathways 

Double Strand Break (DSB) repair is usually carried out via either Homologous Recombination (HR) or Non-Homologous End Joining (NHEJ), following a signal from the S-phase checkpoint. While NHEJ is considered an “error prone” pathway, HR is an “error free” pathway, as it involves repair using a homologous template. Both processes necessitate a 5’ to 3’ resection stage by the proteins of the MRX complex (Mre11, Rad50, and Xrs2) and Exo1 to yield ssDNA, causing also a lowering of histone occupancy at the area of the DSB (reviewed in [79]). In the HR pathway, following the resection, the ssDNA is bound by RPA, which is replaced by Rad51 to create a nucleofilament that will start a search for a homologous template from which to fix the DSB. This response is also mediated by Rad52 and two proteins from its epistasis group, Rad55 and Rad57. Once homology has been found, Rad54 aids in the process of strand invasion, allowing the nucleofilament to form a complex with the double strand. Srs2 is then thought to aid in disbanding the nucleofilament by promoting the dissociation of Rad51 from the ssDNA, so that base pairing can occur and a suitable polymerase can synthesize the strand according to the chosen template (reviewed in [106]). Due to their active participation in the process of HR, accumulation of Rad51 or Rad52, measured by the labeling of these proteins with a fluorescent marker, forms a distinctive dot (focus) that disappear once repair has been completed. Furthermore, accumulation of γ-H2A is considered to be a reliable marker for tracking DNA damage, as it accumulates in the presence of ssDNA and disappears once the checkpoint has been turned off. 

Wild type cells that are exposed to genotoxins such as methylmethane sulfonate (MMS) are still able to replicate their genome following removal of the drug. However, cells deleted for *RTT109* hardly increase their fraction of replicated DNA upon removal of MMS, with regions such as the rDNA direct repeats on chromosome XII being particularly compromised. Moreover, Rad51 foci, which are usually transient in nature, persist for more than nine hours in these cells, suggesting that lack of H3K56Ac impacts a step of homologous recombination downstream to Rad51 [81]. 

On the other hand, *Δhst3 Δhst4* cells, which are hyper-acetylated, depend on Rad52, Srs2, and the MRX complex, that is, on HR, for survival. Oddly, however, *Δhst3 Δhst4* cells are viable in the absence of Rad51, Rad54, Rad55, and Rad57 [52]. These results indicate that in the presence of hyper-acetylation, the entire HR pathway might not be the preferred choice for repairing lesions, but other pathways making use of the MRX complex and Rad52 but independent of Rad51, such as Break Induced Repair (BIR). BIR is a branch of HR repair dealing with the repair of one-ended DSBs, such as those that occur when forks stall and collapse due to the presence of a DNA lesion or a collision with RNA polymerases (reviewed in [107]). In BIR, the broken end is resected, and it invades a homologous chromosome, forming a replicating bubble that copies the entire chromosomal arm until it reaches the telomere. Two branches of BIR were described in the literature, and both depend on Rad52 and the MRX complex. However, one branch is Rad51, Rad54, Rad55, and Rad57 dependent [108] while the other was independent of these proteins but requires Rad59 and Rdh54 [109]. Interestingly, it has been reported that H3K56 acetylation limits extensive repair via the BIR pathway. *Δhst3 Δhst4* cells showed a reduction in BIR frequency, which was restored by expressing H3K56R or by deleting *ASF1* or *RTT109*, showing that H3K56 acetylation is important for a successful repair in situations where only one end is available for invasion [88]. This phenomenon is explained by the fact that hyper-acetylation of H3K56 interferes with replisome stability, and BIR requires the DNA synthesis of many kilobase pairs and sometimes megabase pairs, of DNA.

## 6. Concluding Remarks

Timely acetylation and deacetylation of H3K56 has a profound impact on the ability of cells to accurately respond to DNA lesions and maintain proper DNA replication and repair. The unique location of H3K56 within the nucleosome makes it a key player in shaping the chromatin of the cell, and due to it a complex chain of regulation has evolved to maintain the acetylation on an "as needed" basis only. While this review focuses mainly on replication and genome stability, H3K56 was shown to also play a role in transcription (see [110,111,112] for examples), expression homeostasis [113], silencing (see [114,115] for examples), ribosomal regulation (see [116,117] for examples), telomere biology [118], and aging (see [119,120] for examples). The many roles of this modification were distributed between different posttranslational modifications in mammalian cells. For example, newly synthesized histones are marked by H4K20me0 in mammalian cells [121] while H3K56Ac in these cells is a modification that is prevalent in active promoter regions [122]. Nevertheless, the replication process, and in it the positioning of nucleosomes behind the moving fork, is conserved throughout eukaryotic evolution [123]. This conservation might implicate the acetylation and deacetylation machinery in anticancer treatment. Indeed, dysregulation of sirtuins was shown to have clinical significance and has been observed in many types of tumors, such as breast, prostate, lung, thyroid, liver, colon, gastric, pancreatic, ovarian, and cervical cancers, as well as tumors of the central nervous system, leukemia and lymphoma, and soft tissue sarcomas [124]. This makes pharmacological inhibition of sirtuins a promising avenue cancer treatment. Their targets, as well, are diverse, and include well-characterized housekeeping genes known to be dysregulated in cancer, such as p300, p53, TORC1, and more [124]. These targets, and in particular histone modifications targeted by sirtuins, lead, if unbalanced, to various syndromes. For example, SIRT1 targets MeCP2, and reduced activity of this gene causes Rett syndrome, characterized by mental retardation involving loss of acquired speech. SIRT6 and SIRT7 regulate TNFα, and reduced activity leads to Parkinson’s disease [125]. Despite the vast information available on the many roles of H3K56 acetylation in yeast, there is still much to be discovered in how this, and parallel modifications in mammalian cells, impact the stability of the genome. 

## Figures and Tables

**Table 1:** Phenotypes of *Δhst3 Δhst4* mutants.

Category	Phenotype	Source
Sensitivity to genotoxic agents:	Campthotecin (CPT)	[13]
	Bleomycin (ionizing radiation)	
	Ultraviolet radiation (UV)	[40]
	Methyl Methanesulfonate (MMS)	[41]
	Hydroxyurea (HU)	
	Peroxide (H_2_O_2_)	[51]
Genotoxic stress markers:	Rad52-foci accumulation	[48]
	Ddc2-foci accumulation	
	Spontaneous Rad53 phosphorylation	[52]
Genome stability:	Increased loss of heterozygosity	[54]
	Elevated recombination rates	[55]
	Gross chromosomal rearrangements	
	Small insertions/deletions	
	Elevated rates of nondisjunction events	
Chromatin changes:	Derepression of subtelomeric genes	[56]
	Sister chromatid cohesion defects	[48]
Other:	G_2_/M cycle arrest/delay	[40]
	Thermosensitivity	

## References

[1] Cutter A.R., Hayes J.J. (2015). A brief review of nucleosome structure. FEBS Lett..

[2] Alabert C., Groth A. (2012). Chromatin replication and epigenome maintenance. Nat. Rev. Mol. Cell Biol..

[3] Fillingham J., Recht J., Silva A.C., Suter B., Emili A., Stagljar I., Krogan N.J., Allis C.D., Keogh M.C., Greenblatt J.F. (2008). Chaperone control of the activity and specificity of the histone H3 acetyltransferase Rtt109. Mol. Cell. Biol..

[4] Zou L., Elledge S.J. (2003). Sensing DNA damage through ATRIP recognition of RPA-ssDNA complexes. Science.

[5] Lucca C., Vanoli F., Cotta-Ramusino C., Pellicioli A., Liberi G., Haber J., Foiani M. (2004). Checkpoint-mediated control of replisome-fork association and signalling in response to replication pausing. Oncogene.

[6] Driscoll R., Hudson A., Jackson S.P. (2007). Yeast Rtt109 promotes genome stability by acetylating histone H3 on lysine 56. Science.

[7] Han J., Zhou H., Horazdovsky B., Zhang K., Xu R.M., Zhang Z. (2007). Rtt109 acetylates histone H3 lysine 56 and functions in DNA replication. Science.

[8] Rufiange A., Jacques P.E., Bhat W., Robert F., Nourani A. (2007). Genome-wide replication-independent histone H3 exchange occurs predominantly at promoters and implicates H3 K56 acetylation and Asf1. Mol. Cell.

[9] Peterson C.L., Laniel M.A. (2004). Histones and histone modifications. Curr. Biol..

[10] Davey C.A., Sargent D.F., Luger K., Maeder A.W., Richmond T.J. (2002). Solvent mediated interactions in the structure of the nucleosome core particle at 1.9 a resolution. J. Mol. Biol..

[11] Neumann H., Hancock S.M., Buning R., Routh A., Chapman L., Somers J., Owen-Hughes T., van Noort J., Rhodes D., Chin J.W. (2009). A method for genetically installing site-specific acetylation in recombinant histones defines the effects of H3 K56 acetylation. Mol. Cell.

[12] Ferreira H., Somers J., Webster R., Flaus A., Owen-Hughes T. (2007). Histone tails and the H3 alphaN helix regulate nucleosome mobility and stability. Mol. Cell. Biol..

[13] Masumoto H., Hawke D., Kobayashi R., Verreault A. (2005). A role for cell-cycle-regulated histone H3 lysine 56 acetylation in the DNA damage response. Nature.

[14] Ai X., Parthun M.R. (2004). The nuclear Hat1p/Hat2p complex: A molecular link between type B histone acetyltransferases and chromatin assembly. Mol. Cell.

[15] Verreault A., Kaufman P.D., Kobayashi R., Stillman B. (1996). Nucleosome assembly by a complex of CAF-1 and acetylated histones H3/H4. Cell.

[16] Hyland E.M., Cosgrove M.S., Molina H., Wang D., Pandey A., Cottee R.J., Boeke J.D. (2005). Insights into the role of histone H3 and histone H4 core modifiable residues in Saccharomyces cerevisiae. Mol. Cell. Biol..

[17] Adkins M.W., Howar S.R., Tyler J.K. (2004). Chromatin disassembly mediated by the histone chaperone Asf1 is essential for transcriptional activation of the yeast PHO5 and PHO8 genes. Mol. Cell.

[18] Schwabish M.A., Struhl K. (2006). Asf1 mediates histone eviction and deposition during elongation by RNA polymerase II. Mol. Cell.

[19] English C.M., Adkins M.W., Carson J.J., Churchill M.E., Tyler J.K. (2006). Structural basis for the histone chaperone activity of Asf1. Cell.

[20] Kuo Y.M., Henry R.A., Huang L., Chen X., Stargell L.A., Andrews A.J. (2015). Utilizing targeted mass spectrometry to demonstrate Asf1-dependent increases in residue specificity for Rtt109-Vps75 mediated histone acetylation. PLoS ONE.

[21] Chen S.H., Albuquerque C.P., Liang J., Suhandynata R.T., Zhou H. (2010). A proteome-wide analysis of kinase-substrate network in the DNA damage response. J. Biol. Chem..

[22] Berndsen C.E., Tsubota T., Lindner S.E., Lee S., Holton J.M., Kaufman P.D., Keck J.L., Denu J.M. (2008). Molecular functions of the histone acetyltransferase chaperone complex Rtt109-Vps75. Nat. Struct. Mol. Biol..

[23] Park Y.J., Sudhoff K.B., Andrews A.J., Stargell L.A., Luger K. (2008). Histone chaperone specificity in Rtt109 activation. Nat. Struct. Mol. Biol..

[24] Albaugh B.N., Arnold K.M., Lee S., Denu J.M. (2011). Autoacetylation of the histone acetyltransferase Rtt109. J. Biol. Chem..

[25] Zhang L., Serra-Cardona A., Zhou H., Wang M., Yang N., Zhang Z., Xu R.M. (2018). Multisite Substrate Recognition in Asf1-Dependent Acetylation of Histone H3 K56 by Rtt109. Cell.

[26] Alcasabas A.A., Osborn A.J., Bachant J., Hu F., Werler P.J., Bousset K., Furuya K., Diffley J.F., Carr A.M., Elledge S.J. (2001). Mrc1 transduces signals of DNA replication stress to activate Rad53. Nat. Cell Biol..

[27] Donham D.C., Scorgie J.K., Churchill M.E. (2011). The activity of the histone chaperone yeast Asf1 in the assembly and disassembly of histone H3/H4-DNA complexes. Nucleic Acids Res..

[28] Han J., Li Q., McCullough L., Kettelkamp C., Formosa T., Zhang Z. (2010). Ubiquitylation of FACT by the cullin-E3 ligase Rtt101 connects FACT to DNA replication. Genes Dev..

[29] Zaidi I.W., Rabut G., Poveda A., Scheel H., Malmstrom J., Ulrich H., Hofmann K., Pasero P., Peter M., Luke B. (2008). Rtt101 and Mms1 in budding yeast form a CUL4(DDB1)-like ubiquitin ligase that promotes replication through damaged DNA. EMBO Rep..

[30] Collins S.R., Miller K.M., Maas N.L., Roguev A., Fillingham J., Chu C.S., Schuldiner M., Gebbia M., Recht J., Shales M. (2007). Functional dissection of protein complexes involved in yeast chromosome biology using a genetic interaction map. Nature.

[31] Winkler D.D., Zhou H., Dar M.A., Zhang Z., Luger K. (2012). Yeast CAF-1 assembles histone (H3-H4)2 tetramers prior to DNA deposition. Nucleic Acids Res..

[32] Su D., Hu Q., Li Q., Thompson J.R., Cui G., Fazly A., Davies B.A., Botuyan M.V., Zhang Z., Mer G. (2012). Structural basis for recognition of H3K56-acetylated histone H3-H4 by the chaperone Rtt106. Nature.

[33] Li Q., Zhou H., Wurtele H., Davies B., Horazdovsky B., Verreault A., Zhang Z. (2008). Acetylation of histone H3 lysine 56 regulates replication-coupled nucleosome assembly. Cell.

[34] Yang J., Zhang X., Feng J., Leng H., Li S., Xiao J., Liu S., Xu Z., Xu J., Li D. (2016). The Histone Chaperone FACT Contributes to DNA Replication-Coupled Nucleosome Assembly. Cell Rep..

[35] Nakagawa T., Bulger M., Muramatsu M., Ito T. (2001). Multistep chromatin assembly on supercoiled plasmid DNA by nucleosome assembly protein-1 and ATP-utilizing chromatin assembly and remodeling factor. J. Biol. Chem..

[36] Akey C.W., Luger K. (2003). Histone chaperones and nucleosome assembly. Curr. Opin. Struct. Biol..

[37] Groth A., Rocha W., Verreault A., Almouzni G. (2007). Chromatin challenges during DNA replication and repair. Cell.

[38] Groth A., Corpet A., Cook A.J., Roche D., Bartek J., Lukas J., Almouzni G. (2007). Regulation of replication fork progression through histone supply and demand. Science.

[39] Yu C., Gan H., Serra-Cardona A., Zhang L., Gan S., Sharma S., Johansson E., Chabes A., Xu R.M., Zhang Z. (2018). A mechanism for preventing asymmetric histone segregation onto replicating DNA strands. Science.

[40] Brachmann C.B., Sherman J.M., Devine S.E., Cameron E.E., Pillus L., Boeke J.D. (1995). The SIR2 gene family, conserved from bacteria to humans, functions in silencing, cell cycle progression, and chromosome stability. Genes Dev..

[41] Celic I., Masumoto H., Griffith W.P., Meluh P., Cotter R.J., Boeke J.D., Verreault A. (2006). The sirtuins hst3 and Hst4p preserve genome integrity by controlling histone h3 lysine 56 deacetylation. Curr. Biol..

[42] Maas N.L., Miller K.M., DeFazio L.G., Toczyski D.P. (2006). Cell cycle and checkpoint regulation of histone H3 K56 acetylation by Hst3 and Hst4. Mol. Cell.

[43] Zhu G., Spellman P.T., Volpe T., Brown P.O., Botstein D., Davis T.N., Futcher B. (2000). Two yeast forkhead genes regulate the cell cycle and pseudohyphal growth. Nature.

[44] Delgoshaie N., Tang X., Kanshin E.D., Williams E.C., Rudner A.D., Thibault P., Tyers M., Verreault A. (2014). Regulation of the histone deacetylase Hst3 by cyclin-dependent kinases and the ubiquitin ligase SCFCdc4. J. Biol. Chem..

[45] Ozdemir A., Spicuglia S., Lasonder E., Vermeulen M., Campsteijn C., Stunnenberg H.G., Logie C. (2005). Characterization of lysine 56 of histone H3 as an acetylation site in Saccharomyces cerevisiae. J. Biol. Chem..

[46] Sia R.A., Herald H.A., Lew D.J. (1996). Cdc28 tyrosine phosphorylation and the morphogenesis checkpoint in budding yeast. Mol. Biol. Cell.

[47] Edenberg E.R., Vashisht A.A., Topacio B.R., Wohlschlegel J.A., Toczyski D.P. (2014). Hst3 is turned over by a replication stress-responsive SCF(Cdc4) phospho-degron. Proc. Natl. Acad. Sci. USA.

[48] Thaminy S., Newcomb B., Kim J., Gatbonton T., Foss E., Simon J., Bedalov A. (2007). Hst3 is regulated by Mec1-dependent proteolysis and controls the S phase checkpoint and sister chromatid cohesion by deacetylating histone H3 at lysine 56. J. Biol. Chem..

[49] Irene C., Theis J.F., Gresham D., Soteropoulos P., Newlon C.S. (2016). Hst3p, a histone deacetylase, promotes maintenance of Saccharomyces cerevisiae chromosome III lacking efficient replication origins. Mol. Genet. Genom. MGG.

[50] Madsen C.T., Sylvestersen K.B., Young C., Larsen S.C., Poulsen J.W., Andersen M.A., Palmqvist E.A., Hey-Mogensen M., Jensen P.B., Treebak J.T. (2015). Biotin starvation causes mitochondrial protein hyperacetylation and partial rescue by the SIRT3-like deacetylase Hst4p. Nat. Commun..

[51] Simoneau A., Ricard E., Weber S., Hammond-Martel I., Wong L.H., Sellam A., Giaever G., Nislow C., Raymond M., Wurtele H. (2016). Chromosome-wide histone deacetylation by sirtuins prevents hyperactivation of DNA damage-induced signaling upon replicative stress. Nucleic Acids Res..

[52] Celic I., Verreault A., Boeke J.D. (2008). Histone H3 K56 hyperacetylation perturbs replisomes and causes DNA damage. Genetics.

[53] Fillingham J., Greenblatt J.F. (2008). A histone code for chromatin assembly. Cell.

[54] Andersen M.P., Nelson Z.W., Hetrick E.D., Gottschling D.E. (2008). A genetic screen for increased loss of heterozygosity in Saccharomyces cerevisiae. Genetics.

[55] Kadyrova L.Y., Mertz T.M., Zhang Y., Northam M.R., Sheng Z., Lobachev K.S., Shcherbakova P.V., Kadyrov F.A. (2013). A reversible histone H3 acetylation cooperates with mismatch repair and replicative polymerases in maintaining genome stability. PLoS Genet..

[56] Yang B., Miller A., Kirchmaier A.L. (2008). HST3/HST4-dependent deacetylation of lysine 56 of histone H3 in silent chromatin. Mol. Biol. Cell.

[57] Munoz-Galvan S., Jimeno S., Rothstein R., Aguilera A. (2013). Histone H3K56 acetylation, Rad52, and non-DNA repair factors control double-strand break repair choice with the sister chromatid. PLoS Genet..

[58] Gershon L., Kupiec M. (2021). A novel role for Dun1 in the regulation of origin firing upon hyper-acetylation of H3K56. PLoS Genet..

[59] Dershowitz A., Snyder M., Sbia M., Skurnick J.H., Ong L.Y., Newlon C.S. (2007). Linear derivatives of Saccharomyces cerevisiae chromosome III can be maintained in the absence of autonomously replicating sequence elements. Mol. Cell. Biol..

[60] Kubota T., Myung K., Donaldson A.D. (2013). Is PCNA unloading the central function of the Elg1/ATAD5 replication factor C-like complex?. Cell Cycle.

[61] Shemesh K., Sebesta M., Pacesa M., Sau S., Bronstein A., Parnas O., Liefshitz B., Venclovas C., Krejci L., Kupiec M. (2017). A structure-function analysis of the yeast Elg1 protein reveals the importance of PCNA unloading in genome stability maintenance. Nucleic Acids Res..

[62] Torres J.Z., Schnakenberg S.L., Zakian V.A. (2004). Saccharomyces cerevisiae Rrm3p DNA helicase promotes genome integrity by preventing replication fork stalling: Viability of rrm3 cells requires the intra-S-phase checkpoint and fork restart activities. Mol. Cell. Biol..

[63] Luciano P., Dehe P.M., Audebert S., Geli V., Corda Y. (2015). Replisome function during replicative stress is modulated by histone h3 lysine 56 acetylation through Ctf4. Genetics.

[64] Gambus A., Jones R.C., Sanchez-Diaz A., Kanemaki M., van Deursen F., Edmondson R.D., Labib K. (2006). GINS maintains association of Cdc45 with MCM in replisome progression complexes at eukaryotic DNA replication forks. Nat. Cell Biol..

[65] Gambus A., van Deursen F., Polychronopoulos D., Foltman M., Jones R.C., Edmondson R.D., Calzada A., Labib K. (2009). A key role for Ctf4 in coupling the MCM2-7 helicase to DNA polymerase alpha within the eukaryotic replisome. EMBO J..

[66] Lengronne A., Pasero P. (2014). Closing the MCM cycle at replication termination sites. EMBO Rep..

[67] Moriel-Carretero M., Pasero P., Pardo B. (2019). DDR Inc., one business, two associates. Curr Genet.

[68] Gispan A., Carmi M., Barkai N. (2014). Checkpoint-independent scaling of the Saccharomyces cerevisiae DNA replication program. BMC Biol..

[69] Poli J., Tsaponina O., Crabbe L., Keszthelyi A., Pantesco V., Chabes A., Lengronne A., Pasero P. (2012). dNTP pools determine fork progression and origin usage under replication stress. EMBO J..

[70] Deegan T.D., Diffley J.F. (2016). MCM: One ring to rule them all. Curr. Opin. Struct. Biol..

[71] Green C.M., Almouzni G. (2002). When repair meets chromatin. First in series on chromatin dynamics. EMBO Rep..

[72] Seeber A., Dion V., Gasser S.M. (2013). Checkpoint kinases and the INO80 nucleosome remodeling complex enhance global chromatin mobility in response to DNA damage. Genes Dev..

[73] Weinert T.A., Hartwell L.H. (1988). The RAD9 gene controls the cell cycle response to DNA damage in Saccharomyces cerevisiae. Science.

[74] Pardo B., Crabbe L., Pasero P. (2017). Signaling pathways of replication stress in yeast. FEMS Yeast Res..

[75] Sau S., Kupiec M. (2020). A role for the yeast PCNA unloader Elg1 in eliciting the DNA damage checkpoint. Curr. Genet..

[76] Sau S., Liefshitz B., Kupiec M. (2019). The Yeast PCNA Unloader Elg1 RFC-Like Complex Plays a Role in Eliciting the DNA Damage Checkpoint. mBio.

[77] Downs J.A., Lowndes N.F., Jackson S.P. (2000). A role for Saccharomyces cerevisiae histone H2A in DNA repair. Nature.

[78] Ciardo D., Goldar A., Marheineke K. (2019). On the Interplay of the DNA Replication Program and the Intra-S Phase Checkpoint Pathway. Genes.

[79] Clemenson C., Marsolier-Kergoat M.C. (2009). DNA damage checkpoint inactivation: Adaptation and recovery. DNA Repair.

[80] Hastie C.J., Vazquez-Martin C., Philp A., Stark M.J., Cohen P.T. (2006). The Saccharomyces cerevisiae orthologue of the human protein phosphatase 4 core regulatory subunit R2 confers resistance to the anticancer drug cisplatin. FEBS J..

[81] Simoneau A., Delgoshaie N., Celic I., Dai J., Abshiru N., Costantino S., Thibault P., Boeke J.D., Verreault A., Wurtele H. (2015). Interplay between histone H3 lysine 56 deacetylation and chromatin modifiers in response to DNA damage. Genetics.

[82] Tsabar M., Waterman D.P., Aguilar F., Katsnelson L., Eapen V.V., Memisoglu G., Haber J.E. (2016). Asf1 facilitates dephosphorylation of Rad53 after DNA double-strand break repair. Genes Dev..

[83] Chen C.C., Carson J.J., Feser J., Tamburini B., Zabaronick S., Linger J., Tyler J.K. (2008). Acetylated lysine 56 on histone H3 drives chromatin assembly after repair and signals for the completion of repair. Cell.

[84] Kolodner R.D., Marsischky G.T. (1999). Eukaryotic DNA mismatch repair. Curr. Opin. Genet. Dev..

[85] Hombauer H., Srivatsan A., Putnam C.D., Kolodner R.D. (2011). Mismatch repair, but not heteroduplex rejection, is temporally coupled to DNA replication. Science.

[86] Rodriges Blanko E., Kadyrova L.Y., Kadyrov F.A. (2016). DNA Mismatch Repair Interacts with CAF-1- and ASF1A-H3-H4-dependent Histone (H3-H4)2 Tetramer Deposition. J. Biol. Chem..

[87] Li F., Tian L., Gu L., Li G.M. (2009). Evidence that nucleosomes inhibit mismatch repair in eukaryotic cells. J. Biol. Chem..

[88] Che J., Smith S., Kim Y.J., Shim E.Y., Myung K., Lee S.E. (2015). Hyper-Acetylation of Histone H3K56 Limits Break-Induced Replication by Inhibiting Extensive Repair Synthesis. PLoS Genet..

[89] Sugawara N., Goldfarb T., Studamire B., Alani E., Haber J.E. (2004). Heteroduplex rejection during single-strand annealing requires Sgs1 helicase and mismatch repair proteins Msh2 and Msh6 but not Pms1. Proc. Natl. Acad. Sci. USA.

[90] Chakraborty U., Alani E. (2016). Understanding how mismatch repair proteins participate in the repair/anti-recombination decision. FEMS Yeast Res..

[91] Chakraborty U., Mackenroth B., Shalloway D., Alani E. (2019). Chromatin Modifiers Alter Recombination Between Divergent DNA Sequences. Genetics.

[92] Newman T.J., Mamun M.A., Nieduszynski C.A., Blow J.J. (2013). Replisome stall events have shaped the distribution of replication origins in the genomes of yeasts. Nucleic Acids Res..

[93] Feng B., Lin Y., Zhou L., Guo Y., Friedman R., Xia R., Hu F., Liu C., Tang J. (2017). Reconstructing Yeasts Phylogenies and Ancestors from Whole Genome Data. Sci. Rep..

[94] Theis J.F., Irene C., Dershowitz A., Brost R.L., Tobin M.L., di Sanzo F.M., Wang J.Y., Boone C., Newlon C.S. (2010). The DNA damage response pathway contributes to the stability of chromosome III derivatives lacking efficient replicators. PLoS Genet..

[95] Kovtun I.V., McMurray C.T. (2008). Features of trinucleotide repeat instability in vivo. Cell Res..

[96] Mirkin S.M. (2007). Expandable DNA repeats and human disease. Nature.

[97] Fouche N., Ozgur S., Roy D., Griffith J.D. (2006). Replication fork regression in repetitive DNAs. Nucleic Acids Res..

[98] Pelletier R., Krasilnikova M.M., Samadashwily G.M., Lahue R., Mirkin S.M. (2003). Replication and expansion of trinucleotide repeats in yeast. Mol. Cell. Biol..

[99] McMurray C.T. (2008). Hijacking of the mismatch repair system to cause CAG expansion and cell death in neurodegenerative disease. DNA Repair.

[100] Clemente-Ruiz M., Gonzalez-Prieto R., Prado F. (2011). Histone H3K56 acetylation, CAF1, and Rtt106 coordinate nucleosome assembly and stability of advancing replication forks. PLoS Genet..

[101] Yang J.H., Freudenreich C.H. (2010). The Rtt109 histone acetyltransferase facilitates error-free replication to prevent CAG/CTG repeat contractions. DNA Repair.

[102] Freudenreich C.H., Stavenhagen J.B., Zakian V.A. (1997). Stability of a CTG/CAG trinucleotide repeat in yeast is dependent on its orientation in the genome. Mol. Cell. Biol..

[103] Zhang W., Feng J., Li Q. (2020). The replisome guides nucleosome assembly during DNA replication. Cell Biosci..

[104] Prado F., Clemente-Ruiz M. (2012). Nucleosome assembly and genome integrity: The fork is the link. Bioarchitecture.

[105] Lucchini R., Wellinger R.E., Sogo J.M. (2001). Nucleosome positioning at the replication fork. EMBO J..

[106] Shrivastav M., De Haro L.P., Nickoloff J.A. (2008). Regulation of DNA double-strand break repair pathway choice. Cell Res..

[107] Kramara J., Osia B., Malkova A. (2018). Break-Induced Replication: The Where, The Why, and The How. Trends Genet. TIG.

[108] Malkova A., Naylor M.L., Yamaguchi M., Ira G., Haber J.E. (2005). RAD51-dependent break-induced replication differs in kinetics and checkpoint responses from RAD51-mediated gene conversion. Mol. Cell. Biol..

[109] Signon L., Malkova A., Naylor M.L., Klein H., Haber J.E. (2001). Genetic requirements for RAD51- and RAD54-independent break-induced replication repair of a chromosomal double-strand break. Mol. Cell. Biol..

[110] Feldman J.L., Peterson C.L. (2019). Yeast Sirtuin Family Members Maintain Transcription Homeostasis to Ensure Genome Stability. Cell Rep..

[111] Jung I., Seo J., Lee H.S., Stanton L.W., Kim D., Choi J.K. (2015). Global mapping of the regulatory interactions of histone residues. FEBS Lett..

[112] Minard L.V., Williams J.S., Walker A.C., Schultz M.C. (2011). Transcriptional regulation by Asf1: New mechanistic insights from studies of the DNA damage response to replication stress. J. Biol. Chem..

[113] Voichek Y., Bar-Ziv R., Barkai N. (2016). A role for Rtt109 in buffering gene-dosage imbalance during DNA replication. Nucleus.

[114] Dodson A.E., Rine J. (2015). Heritable capture of heterochromatin dynamics in Saccharomyces cerevisiae. eLife.

[115] Oppikofer M., Kueng S., Martino F., Soeroes S., Hancock S.M., Chin J.W., Fischle W., Gasser S.M. (2011). A dual role of H4K16 acetylation in the establishment of yeast silent chromatin. EMBO J..

[116] Han J., Zhou H., Li Z., Xu R.M., Zhang Z. (2007). Acetylation of lysine 56 of histone H3 catalyzed by RTT109 and regulated by ASF1 is required for replisome integrity. J. Biol. Chem..

[117] Ide S., Saka K., Kobayashi T. (2013). Rtt109 prevents hyper-amplification of ribosomal RNA genes through histone modification in budding yeast. PLoS Genet..

[118] Hiraga S., Botsios S., Donaldson A.D. (2008). Histone H3 lysine 56 acetylation by Rtt109 is crucial for chromosome positioning. J. Cell Biol..

[119] Hachinohe M., Hanaoka F., Masumoto H. (2011). Hst3 and Hst4 histone deacetylases regulate replicative lifespan by preventing genome instability in Saccharomyces cerevisiae. Genes Cells Devoted Mol. Cell. Mech..

[120] Lamming D.W., Latorre-Esteves M., Medvedik O., Wong S.N., Tsang F.A., Wang C., Lin S.J., Sinclair D.A. (2005). HST2 mediates SIR2-independent life-span extension by calorie restriction. Science.

[121] Saredi G., Huang H., Hammond C.M., Alabert C., Bekker-Jensen S., Forne I., Reveron-Gomez N., Foster B.M., Mlejnkova L., Bartke T. (2016). H4K20me0 marks post-replicative chromatin and recruits the TONSL-MMS22L DNA repair complex. Nature.

[122] Lo K.A., Bauchmann M.K., Baumann A.P., Donahue C.J., Thiede M.A., Hayes L.S., des Etages S.A., Fraenkel E. (2011). Genome-wide profiling of H3K56 acetylation and transcription factor binding sites in human adipocytes. PLoS ONE.

[123] Aves S.J., Liu Y., Richards T.A. (2012). Evolutionary diversification of eukaryotic DNA replication machinery. Sub-Cell. Biochem..

[124] Yuan H., Su L., Chen W.Y. (2013). The emerging and diverse roles of sirtuins in cancer: A clinical perspective. OncoTargets Ther..

[125] Mirabella A.C., Foster B.M., Bartke T. (2016). Chromatin deregulation in disease. Chromosoma.

